# A phenomenological model of seizure initiation suggests network structure may explain seizure frequency in idiopathic generalised epilepsy

**DOI:** 10.1186/2190-8567-2-1

**Published:** 2012-01-06

**Authors:** Oscar Benjamin, Thomas HB Fitzgerald, Peter Ashwin, Krasimira Tsaneva-Atanasova, Fahmida Chowdhury, Mark P Richardson, John R Terry

**Affiliations:** 1 Department of Engineering Mathematics, University of Bristol, Bristol, BS8 1TR, UK; 2 Institute of Psychiatry, Kings College London, De Crespigny Park, London, SE5 8AF, UK; 3 College of Engineering Mathematics and Physical Sciences, University of Exeter, Exeter, EX4 4QF, UK; 4 Department of Automatic Control and Systems Engineering, University of Sheffield, Sheffield, S1 3EJ, UK; 5 Sheffield Institute for Translational Neuroscience, University of Sheffield, Sheffield, S10 2TN, UK

## Abstract

We describe a phenomenological model of seizure initiation, consisting of a bistable switch between stable fixed point and stable limit-cycle attractors. We determine a quasi-analytic formula for the exit time problem for our model in the presence of noise. This formula--which we equate to seizure frequency--is then validated numerically, before we extend our study to explore the combined effects of noise and network structure on escape times. Here, we observe that weakly connected networks of 2, 3 and 4 nodes with equivalent first transitive components all have the same asymptotic escape times. We finally extend this work to larger networks, inferred from electroencephalographic recordings from 35 patients with idiopathic generalised epilepsies and 40 controls. Here, we find that network structure in patients correlates with smaller escape times relative to network structures from controls. These initial findings are suggestive that network structure may play an important role in seizure initiation and seizure frequency.

## 1 Introduction

Epilepsy is one of the most common serious primary brain diseases, with a worldwide prevalence approaching 1% [1]. Epilepsy carries with it significant costs, both financially (estimated at 15.5 billion euros in the EU in 2004, with a total cost per case between 2,000 and 12,000 euros [2]) and in terms of mortality (some 1,000 deaths directly due to epilepsy per annum [3] in the UK alone). Further, the seemingly random nature of seizures means that it is a debilitating condition, resulting in significant reduction in quality of life for people with epilepsy.

Epilepsy is the consequence of a wide range of diseases and abnormalities of the brain. Although some underlying causes of epilepsy are readily identified (e.g., brain tumour, cortical malformation), the majority of cases of epilepsy have no known cause [1]. Nonetheless, a number of recognised epilepsy syndromes have been consistently described, based on a range of phenomena including age of onset, typical seizure types and typical findings on investigation including electroencephalography (EEG) [4]. It has been assumed that specific epilepsy syndromes are associated with specific underlying pathophysiological defects.

Idiopathic generalised epilepsy (IGE) is a group of epilepsy disorders, including childhood absence epilepsy (CAE), juvenile absence epilepsy (JAE) and juvenile myoclonic epilepsy (JME), which typically have their onset in children and young adolescents. Patients with IGE have no brain abnormalities visible on conventional clinical MRI, and their neurological examination, neuropsychology and intellect are typically normal; consequently, IGEs are assumed to have a strong genetic basis. At present, clinical classification of IGE syndromes is based on easily observable clinical phenomena and qualitative EEG criteria (for example specific features of ictal spike and wave discharges (SWDs) seen on EEG); whilst a classification based on underlying neurobiology is presently unfeasible. Developing an understanding of epilepsy through exploring the underlying mechanisms that generate macroscale phenomena is a key challenge and an area of very active current clinical endeavour [5].

Epilepsy is a highly dynamic disorder with many timescales involved in the dynamics underlying epilepsy and epileptic seizures. The shortest timescales in epilepsy are those of the physical processes that give rise to the pathological oscillations in macroscopic brain dynamics characteristic of epileptic seizures. For example, the classical SWD associated with absence seizures comprises of a spike of activity in the 20-30 Hz range riding on top of a wave component in the slower 2-4 Hz range, which appears approximately synchronously across many channels of the EEG. These macroscale dynamics are presumably reflecting underlying mechanisms that can rapidly synchronise the whole cortical network. More generally, epileptiform phenomena are commonly associated with activity in the 1*-*20 Hz frequency band, although much higher frequency activity (*>*80 Hz) has been shown to correlate with seizure onset [6].

**Figure 1:**
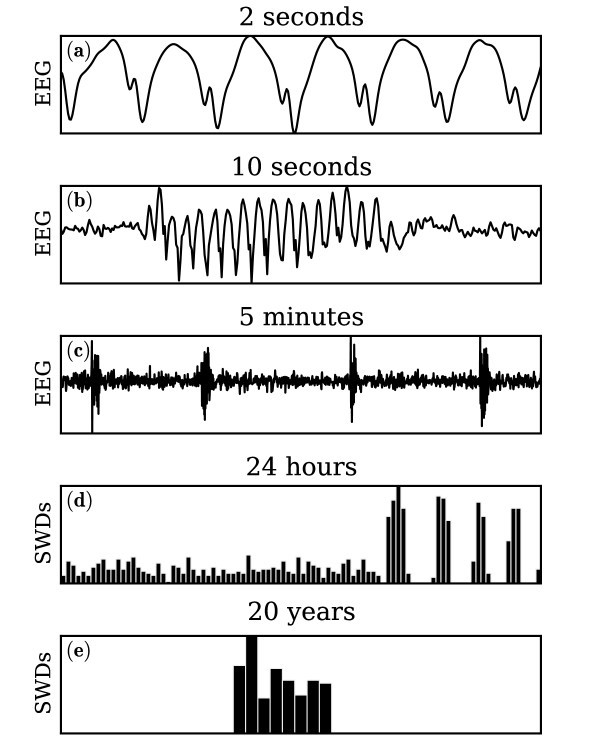
**Timescales in epilepsy and epileptic seizures**. **(a-c) **Consider a single channel of EEG from a patient with absence epilepsy. **(d, e) **Schematics (not based on real data) to illustrate longer timescale phenomena. **(a)**: Close-up of the absence seizure EEG during a SWD showing the spike-and-wave pattern characteristic of absence seizure EEG. **(b)**: Full time course of the same SWD. The epileptiform EEG arises abruptly from the background EEG, oscillates for around 5 **s **(in this case) and terminates abruptly. **(c)**: Period of high SWD activity in a person with absence epilepsy whilst awake. SWD recur almost periodically at around once per minute during this epoch. **(d)**: Schematic histogram of SWD counts during 15-minute epochs over a 24 **h **period. SWD occur steadily throughout waking states and arise in greater number periodically during sleep cycles. **(e)**: Schematic histogram of SWD rates over a 20 **y **period, representing the course of epilepsy within the life of an epileptic person. Absence epilepsy is most likely to start at around age 7. In most cases, after a few years the seizures will have stopped.

The next dynamical timescale is that of the initiation (ictogenesis) and termination of individual seizures, many studies in the field of seizure prediction have shown that changes in macroscopic brain activity in the minutes and hours prior to a seizure may correlate with the likelihood of a subsequent event. Beyond this, there are various circadian factors, for example state of alertness or hormone levels, that can contribute to changes in seizure frequency over timescales of days and weeks. Finally, seizure frequency can vary over a timescale of months and years. For example, children with absence epilepsy typically 'grow out' of the condition upon reaching the early stages of adolescence. We may think of this as the timescale of the *pathology *of epilepsy, or *epileptogenesis*. Ultimately, the fact that a person has epilepsy (unlike the majority of people) is the result of the interaction between several multi-timescale processes and factors. In Figure 1, we present schematically some of the timescales involved in absence seizures and absence epilepsy.

### 1.1 Mathematical models of seizure initiation

In the case of IGE and SWDs in particular, much is known about the physiological processes occurring at short timescales (e.g., ms or s). This is also the timescale characterised by features that are most reproducible across subjects; such as the characteristic SWD that is observed in experimental and clinical EEG recorded during absence seizures.

Some models, such as those of Destexhe [7,8], have extensively analysed the microscopic detail underlying the macroscopic oscillation during SWDs. These models have summarised the detailed *in vivo *evidence regarding the behaviour of individual cells, cell types and brain regions obtained from the feline generalised penicillin model of epilepsy. Taken with more recent *in vivo *data concerning the parametrisation of the various synaptic and cellular currents involved, Destexhe is able to build a complete picture of the oscillations in the context of a microscopic network of thalamocortical (TC) projection, reticular (RE) and corticothalamic (CT) projection cells, along with local inhibitory interneurons in cortex (IN). In this model, SWDs are initiated and terminated by slow timescale currents in TC cells. In between SWDs, all cells are at rest. The rest state of one or two TC cells slowly becomes unstable, however. The initial burst firing of this one cell then recruits the rest of the network, leading to a SWD in the population as a whole. Whilst this model provides excellent insight into the detail of the oscillation, its description of SWD initiation and termination and of inter-ictal dynamics is certainly not applicable to the case of absence seizures occurring during the waking state.

Other models, such as the mean-field model introduced by Robinson et al. [9] and subsequently analysed by Breakspear et al. [10] explicitly separates the short timescale dynamics associated with the oscillatory phase of the SWD from the longer timescales implicated in the initiation and termination of the discharge. In these models, the onset of a seizure results from a dynamical bifurcation of the short timescale dynamics. That is, the model characterises the difference between the inter-ictal and ictal states in terms of a change in parameters rather than a slow change in state space. This model represents the brain in terms of the mean activity of three homogeneous, synchronised cell populations TC, RE, and cortex and enables detailed study of how the relationships between these regions affect the possibility of pathological oscillations. In this context, it is conceived that the brain is at rest (in a macroscopic sense) during the inter-ictal phase and oscillating during ictal activity. The transition between the two states occurs because a parameter of the system changes, resulting in a bifurcation of the resting state. Beyond IGEs, such an approach has also been used to characterise focal seizures, where for example Wendling et al. [11] extended the Jansen and Rit model [12], Grimbert and Faugeras [13] studied bifurcations characterising transitions between dynamics during focal seizures and Liley and Bojak [14] explored systematically varying parameters using anaesthetic agents. Conceptually, however, there is no difference between this approach and that based on slow dynamics. That is, whether or not a transition is the result of slow dynamics or of a change in parameters depends on the choice of timescale for the model; a parameter at a shorter timescale may be considered a dynamical variable at a longer one.

However, there are other candidate mechanisms for seizure initiation. Lopes da Silva [15] proposed that the abrupt transition to ictal activity from background EEG was suggestive of bistability. That is, that both the ictal and inter-ictal states are simultaneously stable in different regions of phase space. In this context, the transition is caused by a perturbation in phase space, from an external input or noisy internal dynamics. Suffczynski et al. [16] then developed a specific model to investigate this mechanism as a way to understand the transition between sleep spindles and SWD. Most recently Kalitzin et al. [17] proposed that stimulation-based anticipation and control of seizures might be possible using a model that is closely related to the one we subsequently introduce. This bistable transition approach is substantially different from the bifurcation hypothesis in the sense that one is driven predominately by stochastic processes, with no substantive changes in underlying parameters over the time course of seizure onset, whilst the other corresponds to a predominately deterministic route to seizures through underlying parameter variation. In practice, both possibilities can occur in the same model, so they are not mutually exclusive [18].

## 2 Building a phenomenological model of seizure initiation

Motivated by clinical observations of synchronised dynamics that occur rapidly across several regions of the cortex, we are interested to explore the role that network structure may play in the *initiation *of a seizure from the inter-ictal state. As exploring this mechanism is our fundamental goal, we do not consider the detailed physiological mechanisms which underlie the 2*-*4 Hz spike-wave dynamics that are the characteristic hallmark of absence seizures observed in EEG. Neither do we consider how processes acting on longer timescales can modulate the instantaneous probability of a seizure event occurring. Instead we assume that the 'excitability' underlying seizure generation is a dynamic constant, so that we may explore the dynamics at the moment of onset of a seizure.

What are the key ingredients that a phenomenological model of seizure initiation should contain? Inspired by the work of Lopes da Silva, we hypothesise here that seizure initiation is a noise-driven process in a bistable system, rather than a result of slower dynamics in a deterministic system. Hence, our model should admit two possible states simultaneously; a resting state (that we consider to be inter-ictal dynamics) and an oscillating state (that we consider to be ictal dynamics). Our choices here are motivated by these being the most prominent features of EEG recorded during these states of activity. Further support for this hypothesis of bistability is found in statistical data from rats and humans with genetic absence epilepsy that indicates seizure initiation is a stochastic process [19]. This study further explores the distribution of inter-ictal intervals and the evidence presented for both GAERS and WAG/RIJ rats is suggestive of a random walk type process for these intervals. Whilst this hypothesis is contrary to many of the studies described earlier--that an external or internal deterministic process triggers the immediate onset of a seizure--these two hypotheses are difficult to distinguish empirically because each represents a dramatic simplification of the physical processes in the real brain. Essentially, our hypothesis reflects our choices of spatial and temporal scales of observation. In reality, the transition between the two macroscopic stable states must be driven by input of some kind. The input most likely arises from a combination of factors including at least external sensory input and the high-dimensional chaos of interactions in the microscopic neuronal networks that make up the brain. To represent these as noise reflects, the fact that the time and space scales we use is too large to consider the detailed activity of individual cells and sensory stimuli.

A further ingredient, since we wish to explore the interplay between topology and seizure initiation, is that our phenomenological model should take the form of a network of interconnected systems. Since we would like to consider the initiation of seizures in the whole brain, consideration of the interaction between distinct cortical regions is an appropriate level of description for the model. Whilst there is considerable evidence of structured networks at the microscale (e.g., interconnected pyramidal (PY)) cells or PY-TC connectivity) or mesoscale (e.g., cortical columns), at the macroscale, TC or cortico-cortical connectivity exhibits very little regularity, repetition or symmetry. Different regions of the brain serve distinct functions, connect to distinct TC relay nuclei, and to other cortical regions without any simple pattern. There is very little geometrical regularity in cortico-cortical connections that could be represented using a rule as simple as *k*-nearest neighbours. Similarly, the continuous symmetric connectivity profiles used in PDE-based models are completely unable to match up with the well-known macroscopic connections of the brain [20]. Consequently, network topologies typically used in modelling neural dynamics are inadequate for our purpose. In the context of our model, we cannot assume that connectivity is either regular or bidirectionally symmetric.

Instead, the formulation we choose reflects the hypothesis that the brain consists of a discrete set of cortical regions, which have irregular directional connectivity. For simplicity, we assume that a connection either exists or does not exist from one region to another and seeks to investigate how the structure of the connectivity affects the properties of the network as a whole. The bistability of the system as a whole is envisaged to arise from the bistability of each individual region. That is, each region in isolation is capable of being either in a seizure state or a non-seizure state, with connections between regions said to be *synchronising*. By this, we mean that if a region *A *has a connection to region *B*, then region *A *will influence region *B*, to behave the same way that region *A *does. So if region *A *is in the seizure state, it will influence region *B *to go into or stay in the seizure state. Similarly, if region *A *is in the non-seizure state, region *B *will be influenced to go into or to remain in the non-seizure state. If regions *A *and *B *are in the seizure state then region *B *will be influenced to have the same phase as region *A*. Within this framework, we do not consider the relative contributions of excitatory or inhibitory connections to this overall synchronising effect.

### 2.1 Equations of motion for a single node

The equations we choose to describe each unit result in a two-dimensional system that exhibits a fixed point and a limit-cycle, both locally attracting. The initial conditions and, more relevantly, the noise realisation will govern which of these two attractors dominate the trajectory of the system at any time. The equations for the deterministic part or at the drift coefficient of the noise-driven system can be expressed as a single complex equation:

(1) $$ż=f\left(z\right)\equiv \left(\lambda -1+i\omega \right)z+2z|z{|}^{2}-\;z|z{|}^{4}.$$

This equation is a special case of a more general form introduced by Kalitzin et al. [17], where the parameter *ω *controls the frequency of oscillation and the parameter *λ *determines the possible attractors of the system. The first two terms on the right-hand side of Equation 1 describe a subcritical Hopf bifurcation with bifurcation parameter *λ*. Without the third term, the system would have a fixed point at *z *= 0, stable for *λ <*1 and unstable for *λ >*1, and an unstable limit-cycle for *λ <*1, with trajectories outside the unstable limit-cycle diverging to infinity. Essentially the third term ensures that the system remains bounded and has an attracting limit-cycle outside the repelling limit-cycle. The precise form of Equation 1, using *λ - *1 instead of simply *λ *and a coefficient of 2 for the second term, is chosen to place the significant features of the system at algebraically convenient locations. The signs of the coefficients ensure that the fixed points and limit-cycles are stable/unstable as required to obtain the region of bistability.

**Figure 2:**
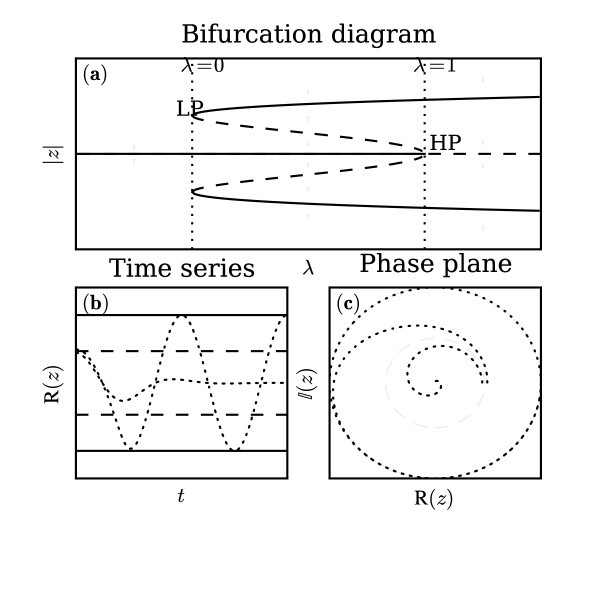
**Dynamics of a single unit**. Graphical description of the dynamical system defined by Equation 1. **(a)**: Bifurcation diagram in the parameter *λ*. The vertical dotted lines mark the parameter values corresponding to the bifurcation points in the system, *λ *= 0 and *λ *= 1. The straight horizontal line represents the fixed point which exists at the origin, *z *= 0, for all values of *λ*. The fixed point is attracting for *λ <*1 (solid line) and repelling otherwise (dashed line). The two pairs of curved lines represent the two limit-cycles in this system. The fixed point becomes unstable at the HP point, HP, at which point the unstable limit-cycle (curved dashed lines) is born. There is also a stable limit-cycle (curved solid lines). The two limit-cycles are joined by a limit-point, LP. **(b)**: Two timeseries (dotted lines) of the system with different initial conditions and 0 *< λ <*1. One trajectory begins just outside the unstable limit-cycle and the other just inside. The two trajectories quickly diverge with one heading towards the fixed point and the other towards the outer limit-cycle. **(c)**: Same two trajectories (dotted lines) in the phase plane. The solid and dotted lines in panels **(b, c) **mark the positions of the stable and unstable limit-cycles, respectively.

We represent the system described by Equation 1, with vector field *f *in panel (a) of Figure 2 as a bifurcation diagram in the parameter *λ*. There is a fixed point represented by the horizontal line, which undergoes a subcritical Hopf (HP) at *λ *= 1, *z *= 0. The curved lines represent the stable $\left(|z{|}^{2}=1+\sqrt{\lambda }\right)$ and unstable $\left(|z{|}^{2}=1-\sqrt{\lambda }\right)$ limit-cycles, which annihilate in a limit-point at *λ *= 0, *|z| *= 1. In summary, the system exhibits three regimes depending on the value of the bifurcation parameter *λ*:

• *λ <*0: The fixed point is stable and globally attracting.

• 0 *< λ <*1: Both the fixed point and the outer limit-cycle are stable and locally attracting. Their basins of attraction are separated by the unstable limit-cycle.

• *λ >*1: The limit-cycle is stable and globally attracting.

For the bistable case, panels (b) and (c) of Figure 2 show two numerically generated timeseries starting just inside and just outside of the unstable limit-cycle. The two series immediately diverge heading towards the fixed point and unstable limit-cycle, respectively.

### 2.2 The interplay between noise and escape time

In the absence of noise, for 0 *< λ <*1, the regions inside and outside of the unstable limit-cycle are *invariant sets*. That is if the initial condition is inside (outside) the unstable limit-cycle, then the trajectory will remain inside (outside) the unstable limit-cycle for all time. More precisely, the trajectories will converge either to the fixed point or to the outer limit-cycle, with the unstable limit-cycle forming the boundary between the basins of attraction of the two attractors.

In the presence of additive noise (which we think of as being due to intrinsic brain dynamics not explicitly considered within our model), a trajectory will (almost surely) leave any region of phase space eventually. We define the noise-driven system using the Itô SDE:

(2) dz(t)=f(z)dt+αdw(t)

where *α *is a constant and *w*(*t*) is a complex Weiner process, equivalent to *u*(*t*) + *iv*(*t*) for two real Weiner processes, *u *and $v\left(i=\sqrt{-1}\right)$. The general dynamics of the system described by Equation 2 depend on the relative size of the deterministic part *f *(the drift coefficient), and the noise amplitude *α *(the drift coefficient). If the noise is large enough, the dynamics will be completely dominated by diffusion. In this case, the system may not spend much of its time near either of the attractors and may cross the boundary between them frequently. When the noise is weak, the system will spend most of its time in the neighbourhood of one or other of the attractors and only occasionally make a large enough deviation that it can cross into the basin of attraction of the other attractor. The larger the noise, the more frequently the trajectory crosses on average.

**Figure 3:**
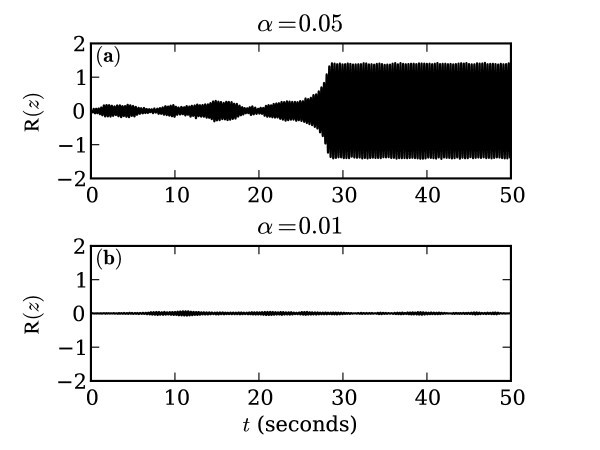
**Example trajectories of two single nodes with *λ *= 0.9 and different values of *α*, the noise amplitude**. In **(a)***α *= 0.05 and in **(b)**: *α *= 0.01.

In Figure 3, we present numerical solutions to Equation 2 for two different values of *α*. The initial condition, *z*(0), is the fixed point (*z *= 0) in both cases but when the noise is larger the system quickly leaves the basin of attraction of the fixed point. The system then stays at the oscillating attractor. The fact that the system leaves the fixed point quickly but then stays near the limit-cycle for long time is due to the imbalance in the strength of the two attractors. For $\frac{1}{4}<\lambda <1$, the limit-cycle is more strongly attracting than the fixed point. Thus, for these values of *λ *(0.9 is used in the figure), the transition occurs much more frequently in one direction than the other. For the other case depicted in Figure 3, the noise is much lower so the system remains near the fixed point for the duration of this simulation. Eventually, however, for both cases, the trajectory will cross from one attractor to the other.

Provided the noise amplitude is non-zero, the probability that a trajectory starting at the fixed point will have made the transition towards the limit-cycle approaches one as the duration of the trajectory increases towards infinity. That is any trajectory will almost surely make the transition to the other attractor eventually. The question then, is not one of whether or not the system will leave the region but how long it takes on average. We quantify this behaviour by identifying the *mean escape time *from the region.

**Figure 4:**
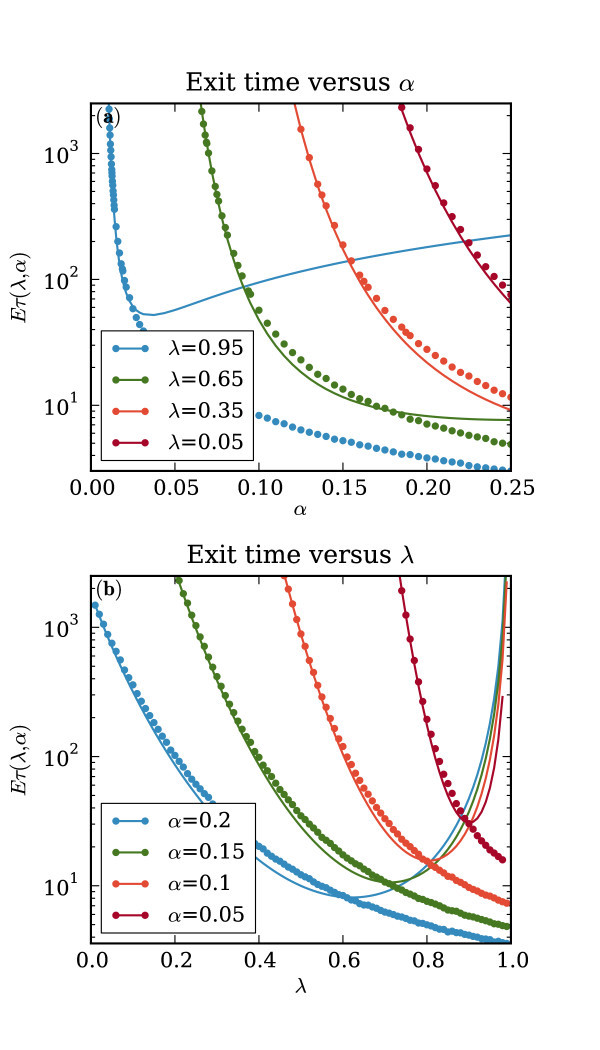
**Distribution of exit times for a fully connected network of 3 nodes, with *α *= 0.05, *λ *= 0.9 and *β *= 1**. The histogram represents numerically obtained escape times from a large number of simulations, normalised to represent a probability density function. The solid line shows an exponential probability density function P(*τ*) = *γ *exp(-*γ τ*) where *γ *≃ 0.007 is the reciprocal of the mean of the numerically obtained escape times. The histogram is well matched by the exponential distribution apart from at short times. This is because the initial conditions *z *= 0 make it difficult for the system to instantly jump over the escape barrier. These data suggest that the escape process is Poissonian except for at very short times.

Formally, there is a fixed point at the *z *= 0, which is attracting within the region bounded by the unstable limit-cycle. The exit problem corresponding to the transition between the two states is, then, as follows. If a system obeying Equation 2 has initial condition *z*(0) = 0, what is the expected escape time, E[τ], until the system crosses the repelling boundary defined by $|z{|}^{2}<1-\sqrt{\lambda }$. Here, the expectation operator, E[.], refers to the expectation over the distribution of the noise. Figure 4 shows the distribution of escape times for a particular set of parameters obtained numerically. Since the distribution of escape times is, apart from at very small times, exponential, it can be characterised simply by its expected value.

Recall that we consider the stable fixed point of the vector field, *f*, as corresponding to the waking, non-seizure (inter-ictal) brain state. Similarly, the stable limit-cycle is representative of the ictal (seizure) state. Consequently, transitions between these two are interpreted as representing the initiation and termination of seizures. In this interpretation, then, the expected time until the transition from the basin of attraction of the fixed point is directly related to the duration of the interval between seizures or inversely related to the frequency of seizure occurrence.

To understand how the mean escape time, E[τ], varies as a function of model parameters, we consider both numerical and approximate analytic results for comparison. The numerical results come from calculating the sample mean of the escape times from a large number of numerically generated trajectories. Since the parameter *ω *has no effect on E[τ], there are only two parameters to consider: the noise amplitude *α *and the excitability parameter *λ*. Trajectories in the exit problem all begin at the fixed point where the linear term in *f *dominates. Thus, *λ - *1 represents the stability of the stable fixed point. Conversely, we can think of *λ *as representing the *excitability *of the system. As *λ *→ 1, the system becomes more excitable and the expected escape time E[τ]→0 implying that all trajectories head towards the stable limit-cycle instantly. Since the deterministic part of the system, *f*, can be written in terms of the gradient of a potential function, *ψ*(*z*), we can write an approximate analytical formula for the escape time (see appendix A for details). The resulting expression

(3) $$E\left[\tau \right]≃\frac{\sqrt{\pi }\alpha \text{exp}\left(2\frac{\hat{\psi }\left(\lambda \right)}{\alpha^{2}}\right)}{2\sqrt{2}\lambda^{\frac{1}{4}}\left(1-\sqrt{\lambda }\right)\left(1-\lambda \right)},$$

which is asymptotically valid for small *α*, describes the escape time in terms of the potential difference $\hat{\psi }\left(\lambda \right)$ between the fixed point and the lowest part of the repelling escape boundary which is given by

$$\hat{\psi }\left(\lambda \right)=\frac{1}{6}-\frac{1}{2}\lambda +\frac{1}{3}\lambda^{\frac{3}{2}}.$$

**Figure 5:**
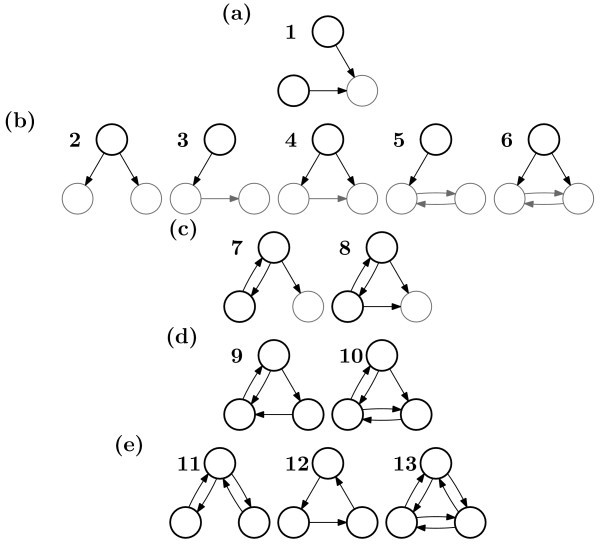
**Escape times: comparison of numerical and analytic results**. Comparison of escape time results from numerical simulation (points) and Equation 3 (dotted lines). **(a)**: Escape time as a function of *α *for different values of *λ*. **(b)**: Escape time as a function of *λ *for different values of *α*.

To consider the validity of this approximate analytic result, we compare it with numerical results in Figure 5. It can be seen that for large escape times (ℰ[*τ*] ≥ 100) both sets of results are in close agreement over a broad range of values. However, it must be noted that the results diverge either as *λ *→ 1 or as *α *becomes large. We can justify this discrepancy qualitatively as follows.

As *λ *→ 1, Equation 3 predicts that the escape time, ℰ[*τ*] → ∞. This is clearly incorrect since the escape boundary is the unstable limit-cycle. Thus as *λ *→ 1, the region from which the trajectory must escape is shrinking towards the fixed point (see Figure 2). As the boundary shrinks towards the initial condition of the escape problem, the escape time must tend towards zero, unless the vector field, *f*, becomes larger in magnitude. However, since each term in the vector field is proportional to a positive power of *z*, as the escape boundary shrinks towards the fixed point, the maximum magnitude of the vector field within the escape region tends towards zero. Consequently, as *λ *→ 1, we must have that ℰ[*τ*] → 0.

Similarly, Equation 3 predicts that the escape time will be a decreasing function of the noise amplitude, *α*, when *α *is small, but an increasing function when *α *is large. However, as the noise amplitude, *α*, becomes larger, the system escapes the potential well sooner. In other words, as *α *→ ∞, we again have that ℰ[*τ*] → 0.

In both cases, the divergence between the two sets of results in Figure 5 is due to a failure of the assumptions in the *analytical *result. The close agreement between the two results at other parameter values is good enough to validate the numerical results and to obtain a qualitative understanding of how the escape time varies. We conclude that, broadly, the mean escape time varies exponentially in the potential barrier and that it is smooth and monotonically decreasing in both *λ *and *α*. When the noise amplitude *α *increases, or as the excitability parameter *λ *→ 1, the mean escape time decreases, or the 'seizure rate' increases.

## 3 A network model

We now generalise the model described above to the case of a coupled network of *N *nodes. The system describes *N *nodes, with 1 complex equation (two-dimensions) each. The system of SDEs we consider is described by:

(4) $$\text{d}z_{i}\left(t\right)=\left(f\left(z_{i}\right)+\beta \underset{j\neq i}{\sum }M_{ji}\left(z_{j}-z_{i}\right)\right)\text{d}t+\alpha \text{d}w_{i}\left(t\right),$$

where **M **is a normalised adjacency matrix, *β *is the coupling strength between connected node and *f *is as defined in Equation 1. The matrix, **M**, is defined such that **M***_ij _*is 1 if there is a connection *from *the *i*th unit *to *the *j*th unit, and zero otherwise. The directionality of the connection is such that a non-zero **M***_ij _*means that the state of the *i*th node, *z_i_*, influences the state of the *j*th node, *z_j_*. Equation 4 treats coupling between connected nodes as linear and simply proportional to the difference between the states of the two nodes. In networks characterised by bidirectional connectivity, this is known as *diffusive coupling*. Further, in generalising to the network case, we have made the assumption that the *i*th node receives noisy input from its own Weiner process, *w_i_*(*t*), independently of the other nodes but with the same noise coefficient *α*. The five parameters of the network model, with typical range of values, are presented in Table 1. We refer the interested reader to [17], where the dynamics of the system are considered for a range of parameter choices.

**Table 1:** Descriptions of model parameters and typical values

Parameter name	Typical values	Description
*ω*	20 rad/s	Oscillatory frequency (approximately 3 Hz)
*α*	0.01-0.20	Noise amplitude
*λ*	0-1	Excitability parameter
*β*	0.01-1	Coupling strength
**M**		Adjacency matrix, topology of network

The exit problem is independent of *ω *for the case of the homogenous network we consider. The chosen value is to mimic the approximately 3 Hz oscillations that are characteristic of SWD. As previously, increases in either *α *or *λ *reduce the escape time all else being equal. Since an increase in either parameter can be compensated for by a decrease in the other, we do not consider the full parameter space. Any specific combination of the two will define the excitability of the network independently of any of the network properties. Since we are interested in the interplay between network structure and escape time, our strategy will be to choose particular values for these two in order to compare how changes in the network properties affect the system, all else being equal.

The parameters of interest, then, are *β *and **M **that respectively define the strength and the topology of the couplings in the network. The topology of the network is defined by its connectivity graph, or equivalently by its adjacency matrix, **M**. Connections are not required to be bidirectional (e.g., **M **need not be symmetric). Finally, since each of the nodes in the network is identical, they are interchangeable. This means that the class of graphs describing the networks considered here is the class of *directed, unweighted, unlabelled *graphs. For networks of *N *nodes, this class is finite, which permits us to consider how the escape time varies as a function of *β *for each possible **M**.

### 3.1 Two-node networks

**Figure 6:**
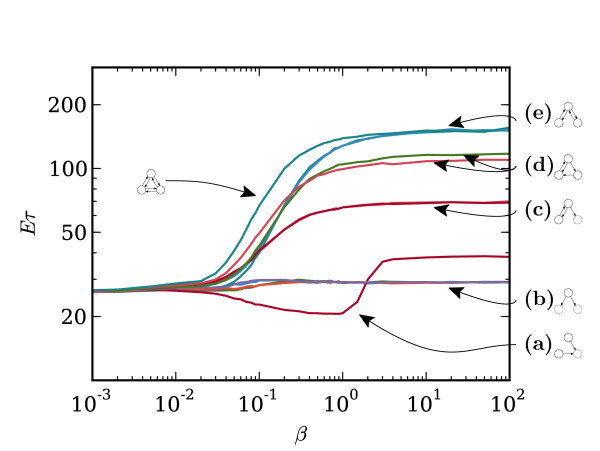
**Networks of two nodes**. **(a) **Presenting the three topologically distinct networks consisting of two nodes. A two-node network may either be fully disconnected, weakly connected or fully connected. **(b) **Escape time ℰ[*τ*] as a function of *β *for the three different two-node networks. **(c) **Reduced phase space for the disconnected two-node network (depicted top middle), with *λ *= 0.5 and *β *= 0.1. This space is spanned by *r_A _*and *r_B_*, the Cartesian distance of the state of each node (*z_A _*and *z_B_*), from the fixed point. Quiver arrows show the direction and magnitude of the vector field within this space. Circles show attracting fixed points within this space. Outset plots show timeseries of the full system (with *ω *= 20) corresponding to the fixed points (circles) of the reduced space (*x_A _*and *x_B _*are displaced vertically for clarity). The solid lines mark the boundaries of the basins of attraction of the fixed points. The dotted line shows a noise-driven trajectory in the full space with *α *= 0.2 and initial condition *z_A _*= *z_B _*= 0. The lower-left circle is the origin of the full space which is a fixed point. The other three circles correspond to attracting limit-cycles, in which either one or both of the nodes is at the limit-cycle. **(d) **Reduced phase space for the weakly connected two-node network (depicted top middle). **(e) **Networks of two nodes: fully connected network. Presenting the reduced phase space for the fully connected two-node network (depicted top middle).

Initially, we consider the simplest networks: those consisting of only two nodes. Figure 6a shows the three distinct graphs in this scenario. In what follows we shall consider the phase space of each network in turn. Since the phase space of each node is two-dimensional, each network is a 4-D system. To consider this graphically, we represent them in the form of a *reduced *phase space. To do this, we convert the equations for each node to polar coordinates and assume that the phase, *θ*, of the two nodes is equal. Consequently, we consider the dynamics of the system defined in terms of *r*_1 _and *r*_2_, where *r_i _*= |*z_i_*|. The fixed points within this space are the points such that the *r_i _*will remain constant over time, implying either a steady state or a limit-cycle in the full phase space.

The first network is *disconnected*; there are no connections between any of the nodes. It is instructive to consider this degenerate case since in the limit of weak connections (*β *→ 0), any graph becomes equivalent to a fully disconnected graph. Figure 6c represents the reduced phase space of this network, which has four attractors. The two synchronised attractors are the states in which both nodes are either at the steady state (*z_A _*= *z_B _*= 0), or the limit-cycle $\left(|z_{A}{|}^{2}=|z_{B}{|}^{2}=1+\sqrt{\lambda }\right)$. The other two attractors correspond to the cases where one node is at rest and the other is oscillating and vice versa. Since the two nodes are uncoupled, their dynamics are independent and transitions between the two states can occur independently for each node.

The second network is the *weakly connected *network which has a single connection from *A *to *B *(equivalently from *B *to *A *by interchangeability). In the weakly connected network, the evolution of node *B *is affected by the state of node *A *but the converse is not true. If *β *is strong enough (0.1 in this case), then the full system will not have a stable attractor in which node *A *is oscillating whilst node *B *remains at the fixed point. In the limit as *β *→ 0, we recover the disconnected graph, so for smaller values of *β*, the system will have the four possible attractors again. If *β *were made much larger, then we would observe only the two synchronised attractors. The trajectory shown here makes a transition to the right and hovers in the vicinity of where the (now non-existent) unsynchronised state would be, before converging towards the synchronised oscillatory state at the top right. Thus, although the deterministic system does not have a fixed point in the bottom-right corner, the noise-driven trajectory may still in some sense be attracted to this part of phase space. Starting from the fixed point, trajectories for this network are more likely to make the transition to the right and then upwards than the other way around (see Figure 6d).

The final two-node network (Figure 6e is the *strongly connected *network. It has two connections, one from *A *to *B *and one from *B *to *A*. Thus, the evolution of both nodes is affected by the state of the other. The network is symmetric, as was the disconnected network (above), but this time the dynamics of the two nodes are not independent. As a result, the boundaries between the attractors are distorted into curves and the unsynchronised attractors actually correspond to oscillations of different amplitude. It is easy to see how this phase space will be gradually deformed into that of Figure 6c as *β *→ 0.

Since for most values of *λ*, the oscillating state is more strongly attracting than the other attractors, virtually all trajectories will end up in the state in which all nodes are oscillating. Provided all nodes are connected and *β *is not very small, trajectories in which a node makes the transition to the oscillating state and back again before another node makes the transition at all are rare. Thus, it still makes sense to think of the whole network as having undergone a transition with an associated escape time. However, since not all nodes in the network begin oscillating at *exactly *the same time, we need to define the escape time for a trajectory of a network. The definition of escape time we will use for the network is that the escape has occurred when at least half of the nodes in the network have made the transition to the limit-cycle.

Figure 6b shows numerical results for how the escape time depends on *β *for each of the three two-node networks described above and for the choices of *λ *= 0.9 and *α *= 0.05. The vector field for the disconnected network is independent of *β *and consequently its escape time is independent of *β *as well. As expected, in the limit of weak coupling, as *β *→ 0, the escape times for all three networks converge. For intermediate values of *β*, the escape time is an increasing function of *β*. For large values of *β*, the escape times converge to a value that no longer depends on *β*. We further note that the order of the escape times between the three different networks is preserved and consistent with saying that a network with more connections has a greater escape time. For different values of *α *and *λ*, the escape times are scaled up or down. However, the qualitative features of the plot and in particular the ordering of the three different networks remain unchanged. From our preliminary study of two-node networks, it appears that having more connections make the network more stable around the region of the steady state, thus making it harder for the transition to the limit-cycle (notionally to ictal dynamics) to occur.

### 3.2 Three-node networks

**Figure 7:**
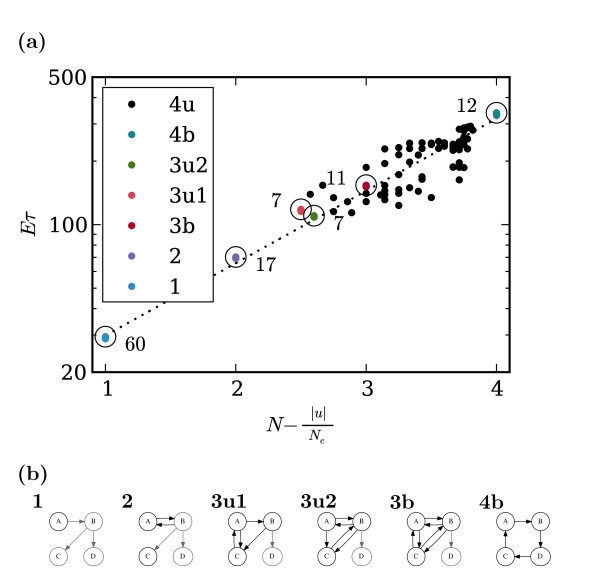
**Networks of three nodes**. Graphical representation of the thirteen topologically distinct networks of three nodes. Networks are grouped according to their escape times in the limit *β *→ ∞ (see Fig. 8). Node and edge colours are used to highlight the *first component *of the network (black) as distinct from the rest of the network (grey). For the weakly connected networks (**a, b, c)**, the first component controls the dynamics of the network as a whole. For the strongly connected networks **(d, e)**, the first component is the whole network. **(a)**: Network **1 **has one node receiving an input from each of the other nodes. The two black nodes are the first component of the graph but they are themselves disconnected. **(b)**: Networks **2-6 **each have a single node in the first component. **(c)**: Networks **7 **and **8 **each have a strongly connected two-node graph as their first component. **(d)**: Networks **9 **and **10 **are strongly connected but unbalanced. The number of inputs and outputs are not equal for each individual node. Note that the escape times for these two networks are similar but, unlike those in the other groups, do not converge in the limit *β *→ ∞ (see Figure 8). **(e)**: Networks **11-13 **are the strongly connected, balanced networks. For each node, there are as many inputs as outputs.

**Figure 8:**
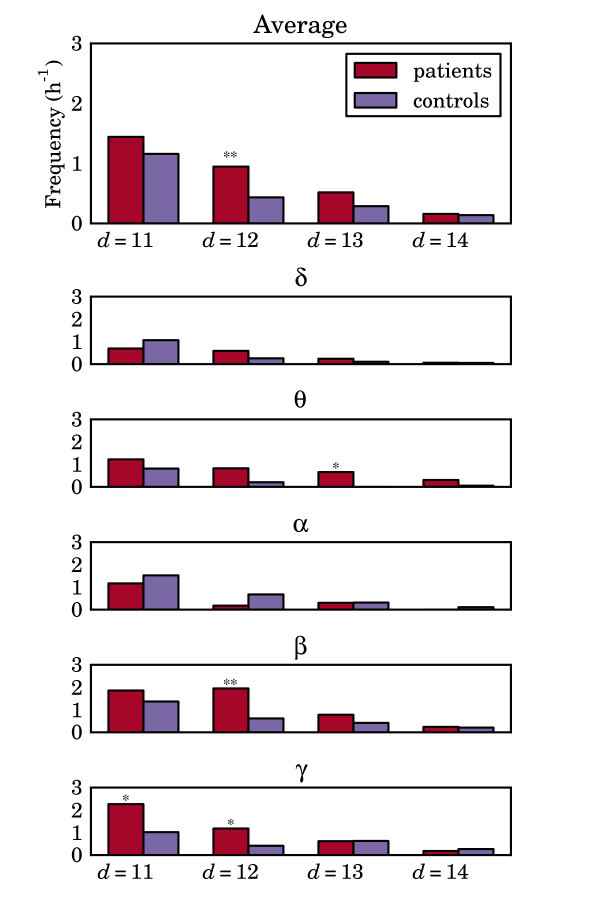
**Networks of three nodes: escape times**. Escape time, ℰ[*τ*], against connection strength, *β*, for the 13 different three-node networks depicted in Figure 7, with *λ *= 0.9 and *α *= 0.05. As *β *→ 0, the escape times for all networks converge to a common value since in this limit all networks become equivalent to the fully disconnected network. In the other direction, as *β *→ ∞, we find that there are groups of networks whose escape times appear to converge to a common value for the group. These groups are labelled **(a-e) **corresponding to the similarly labelled groups in Fig. 7, and in each case an example network from the group is depicted alongside the label. It appears that there is in some sense an equivalence between the networks within each of these groups that manifests in common behaviour when the connection strength is strong. For intermediate value of *β *(~ 0.01-1.0), the escape time moves smoothly between its value at the two extremes, for most networks. The exception is network **1 **(group **(a) **which has the lowest escape time for *β <~ *2.0, but a higher value for *β >~ *2.0. This is due to the fact that this network has a disconnected first component. For this particular network, the qualitative behaviour depends on the general parametrisation of the system. The fully connected network (network **13**) has the highest escape time for all values of *β*.

The next simplest case is that of networks consisting of three nodes. Figure 7 shows the set of 13 topologically distinct networks consisting of three nodes that are at least weakly connected. The escape times for each of these networks are shown in Figure 8. Again, we find that for *β *→ 0, the escape times for all networks converge to a common value. However, what is most striking about this plot, is that, at large values of *β*, the escape times appear to converge into distinct groups. In some sense, it appears that, for strong coupling, some networks are equivalent to each other in terms of the exit problem.

Those networks with more connections generally have higher escape times and are thus more stable around the fixed point. This makes intuitive sense as diffusive coupling will tend to stabilise the network.

However, unlike the case of two-node networks, it is apparent that the ordering of the networks is not wholly consistent with the simple statement "the escape time increases with the number of connections." Moreover, those networks falling into groups with the same escape time do not necessarily have the same number of connections.

One feature that is clear is that all weakly connected networks have lower escape times than all strongly connected networks. Among the weakly connected networks, the grouping appears to occur according to the *first transitive component *(FTC) of the graph defining the topology of the network. This is particularly clear as *β *→ ∞. Figure 7 illustrates what is meant by the FTC by showing the corresponding nodes and edges black, instead of grey. A formal definition for the FTC of a graph is as follows.

Consider a directed graph, **G**. For each distinct pair of nodes *A *and *B *in **G**, we say that *A *≪ *B *if there exists a directed path from *A *to *B *within **G**. The FTC is the set of all nodes *A *such that any *B *that satisfies *B *≪ *A *also satisfies *A *≪ *B*. Equivalently, we define the FTC in terms of its complement in G; the set of nodes that are *not *in the FTC are the nodes *B *such that there is a node *A *with *A *≪ *B *and B≪̸A. For strongly connected graphs, the FTC is the whole graph. In most cases, the FTC of a graph is a strongly connected component. In some cases, however, such as graph **1 **in Figure 7, the subnetwork corresponding to the FTC, as defined here, is a disconnected graph. The FTC, by definition, cannot be weakly connected. Where the FTC is disconnected, network transitions may not be synchronous and the definition of the time of transition becomes somewhat arbitrary, since it is possible that for long periods of time some nodes are in the oscillatory state whilst others are still in the resting state. Thus, it is only really possible to unambiguously define the escape time in cases where the FTC is strongly connected, which is the case that we consider in more detail below.

Whilst this explains differences within weakly connected networks and between weakly connected networks and strongly connected ones, what this does not explain is why strongly connected networks (the top three groups in Figure 8) do not have the same escape times. It appears, in some sense, that the three networks with the highest escape times (11, 12, and 13) are more balanced than those that have lower escape times (9 and 10). Though the only two bidirectional networks (11 and 13) are in the highest grouping, so also is network 12, which is not bidirectional. One way to summarise these three networks is to say that they are the only networks whose edge sets are composed of a union of disjoint cycles. Another way to differentiate them from 9 and 10 is to say that these networks are the ones in which each node has the same number of outgoing as incoming connections. In appendix B, it is shown that the deterministic movement of the centre of mass of the network is determined by the projection of the state of the system onto a vector **u**, of dimension *n *(the size of the network), where **u***_i _*is equal to the out-degree of node *i *minus the in-degree of node *i*. This seems like a relevant quantity here since this vector will be the zero vector for networks 11, 12 and 13 but not for networks 9 and 10. This may be a way to predict the differences between the five strongly connected networks shown in Figure 8.

### 3.3 Networks with four or more nodes

**Figure 9:**
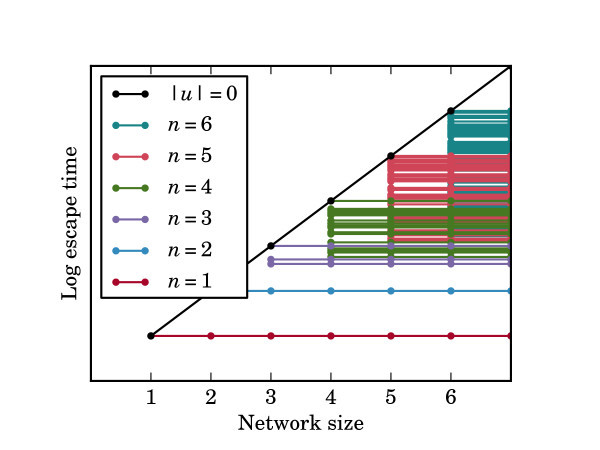
**Networks of four nodes: escape times**. **(a)**: Comparison of Equation 5 with numerical escape time data for four-node networks. Each point represents one topologically distinct network. The vertical axis represents the escape time, E[τ], for this network with large *β *(from simulations using *β *= 100). The horizontal axis represents the expression $N-\frac{\left|u\right|}{N_{e}}$(see Equation 5). Since the vertical scale is logarithmic the apparent straight line relationship suggests an exponential relationship as predicted in Equation 5. The dotted line is a fit, with equation *y *= exp(*ax *+ *b*). Like the right-hand side of Fig. 8, there are groupings of networks having near identical escape times. Graphs from a few groups are plotted as coloured dots. Since these data-points are very close together, they are hard to distinguish visually. Instead each group is circled and the number of graphs within the group is indicated. The remaining 71 graphs are shown as black dots. **(b)**: Example graphs to illustrate the groups identified in **(a)**. Groups of graphs are distinguished by their FTC and whether or not they are *balanced *or *unbalanced*. A *balanced *graph is a graph in which each node has the same number inputs as outputs (in which case |**u**| = 0). **1**: This is a group of 60 graphs each of which has a single node as its FTC, like those in Fig. 7b. **2**: 17 graphs each having a 2-node strongly connected network as its FTC, (Fig. 7c. **3u1**: 7 graphs each having a particular 3-node graph (**9 **from Fig. 7) as its FTC. **3u2**: 7 graphs each having a particular 3-node graph (**10 **from Fig. 7) as its FTC. **3b**: 11 graphs each of which has a balanced 3-node graph as its FTC (in a *balanced *graph, each node has the same number of inputs as outputs, i.e., |**u**| = 0). **4b**: 12 strongly connected graphs each of which is balanced.

**Figure 10:**
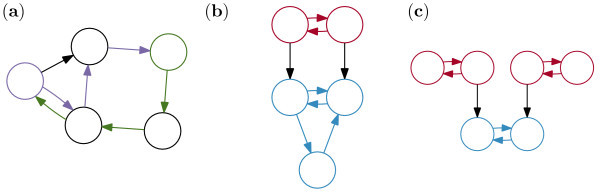
**Escape times for networks of different sizes**. Schematic illustration of how escape times scale with the size (number of nodes) in the network depending on the nature of the FTC when there are strong connections (large *β*). The diagonal black line represents balanced networks (those with **u **= 0). The horizontal lines represent networks of different sizes having the same FTC, coloured according to the number of nodes in the FTC, as specified in the legend. Dots indicate actual graphs and lines are used to convey visually the link between graphs of different size that have similar properties. For the class of balanced networks (**u **= 0) which includes all fully connected and symmetric networks among others, the escape time scales exponentially with the number of nodes in the network. This class of networks also has the largest escape times among networks of a given size. The horizontal lines represent networks of a given FTC, coloured according to the number of nodes in the FTC, *n*. As the network grows in size, provided the FTC is unchanged and the connections are strong, the escape time will remain practically unchanged, leading to the horizontal lines depicted here. For each of *n *= 1 and *n *= 2, there is only one possible FTC. As *n*, the size of the FTC increases beyond two the number of topologically distinct FTCs increases. This is reflected in the increasing number of distinct lines as *n *increases.

To be able to confirm a relationship between **u **and E[τ], there is insufficient data contained in these 13 three-node networks. For this reason, we further extend our analysis to include all 216 topologically distinct four-node networks. For this case, we again find that the escape time of a network is well predicted from its FTC by the relation:

(5) $$E\left[\tau \right]\sim \text{exp}\left(N-\frac{\left|u\right|}{N_{e}}\right),$$

where *N *is the number of nodes (in the first strongly connected component), **u **is the vector of differences between in-degree and out-degree of the nodes and *N_e _*is the number of edges. Figure 9 shows a line fit to the log escape times using an expression of the form in Equation 5. Figure 10 illustrates schematically how ℰ[*τ*] scales with the network size *N*.

### 3.4 Brain networks

Our quasi-analytic results demonstrate a clear relationship between network structure and the mean escape time--which we think of as seizure frequency--in our phenomenological model. This relationship that strongly connected networks demand a greater escape time, all else being equal, than weakly connected networks and the relationship to the first strongly connected component warrants further investigation in larger networks that might be more representative of those present in the human brain. The rate of expansion of distinct network types for a network of size *N *precludes us from considering this question analytically, so instead we consider a different approach. From a database of EEG recordings from 35 patients presenting with IGE and 40 healthy controls, twenty-second epochs (free of ictal discharges and other artefacts) were extracted. In each case, these epochs were bandpass filtered into five distinct frequency bands: *δ*, *θ*, *α*, *β *and *γ *and the level of phase synchrony within each band was calculated pairwise for all 19 electrodes, using the phase-locking factor (PLF). The PLF is a measure of phase synchrony between two digitally sampled signals that is derived from the discrete Hilbert transform and is defined in appendix D.

By assuming that the resulting 19 × 19 matrices of PLF factors, **M***_x,y_--*where *x *is the frequency band and *y *the subject identifier-could be interpreted as a Pearson correlation matrix, a directed graph was then inferred as follows. To measure the 'strength' of a connection from channel *i *to channel *j*, we use the regression coefficient for channel *i *in predicting channel *j*. However, since the regressions coefficient also depends on the amplitude of the signal in both channels, we in fact used the *normalised *regression coefficient, or *β*-weight. Given a Pearson correlation matrix **P **between a set of variables, the matrix of *β*-weights between all variables can be computed from **R **= **P^-^**^1 ^by *β_ij _*= -*R_ij_/R_ii_*. These weights give an effective measure of the *directed *contribution to the total correlation between the *i*th and *j*th node.

To convert the matrix *β *of *β*-weights into a topological adjacency matrix, we applied a threshold to the absolute value of the elements of the matrix. The threshold was chosen to obtain a graph with a specified mean degree *d *per node, where *d *≤ 18 (one less that the number of nodes (EEG channels)). By this, we mean that *d *= 10 implies a network with 19 nodes has 190 edges. We found that, from these particular phase synchrony matrices, the mean number of edges required to guarantee that all graphs were weakly connected was *d *≥ 11, whilst *d *≥ 13 was required to ensure strong connectivity. Whilst assuming the matrix **M **to be equivalent to a correlation matrix is not a mathematically valid assumption (since all correlation matrices have the additional constraint of being positive semidefinite), it is a practical way of constructing a directed graph.

**Figure 11:**
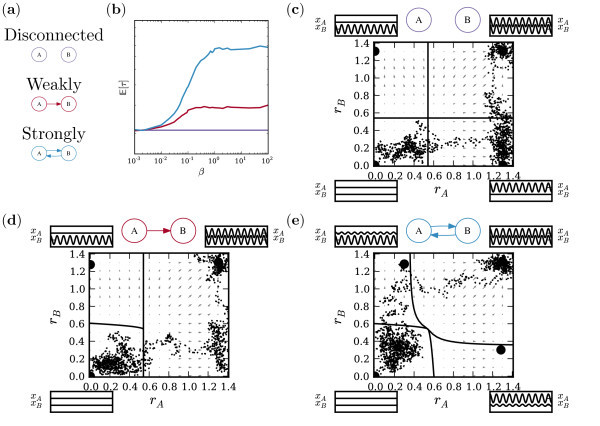
**Seizure frequency for patients and controls, using different values of the mean degree graph parameter, *d***. The bottom five panels are based on graphs obtained from the phase synchrony matrices of the frequency bands, *δ*, *θ*, *α*, *β*, and *γ*. The top panel is based on the average over all frequency bands, for each subject. Bars represent the average seizure frequency over all subjects within each group in units of seizures per hour. Corresponding pairs of seizure rates for patients and controls were compared using the Wilcoxon rank sum test, with normal approximation, according to test hypothesis that patients have higher seizure rates than controls. Statistically significant differences are marked with a single asterisk (*p <*0.05) or a pair of asterisks (*p <*0.01). Rates are computed using simulations with *β *= *α *= 0.1.

From each matrix **M***_x,y_*, networks with mean number of edges *d *= 11, 12, 13, 14 were considered and numerical simulations performed with network parameters *β *= *α *= 0.1. From these simulations, we estimated the number of transitions per hour from the steady state to the limit-cycle (as a proxy for seizure frequency). The findings of our analysis (presented in Figure 11) show a consistent trend when averaging across all frequency bands, in that there is a higher "seizure frequency" (e.g., lower escape time) in those networks calculated from the EEG of patients, relative to those calculated from the EEG of controls. Comparing these differences across the patient and control groups using a one-sided Wilcoxon rank sum with normal approximation, we see that for *d *= 12, the difference is statistically significant (*p <*0.01). Breaking this average down into individual frequency bands presents a more mixed picture, with the observation most dominant in the *β *and *γ *frequency bands; for *d *= 12 statistical significance in *β *(*p <*0.01) and *γ *(*p <*0.05), and for *d *= 11 statistical significance in *γ *(*p <*0.05).

## 4 Discussion

**Figure 12:**
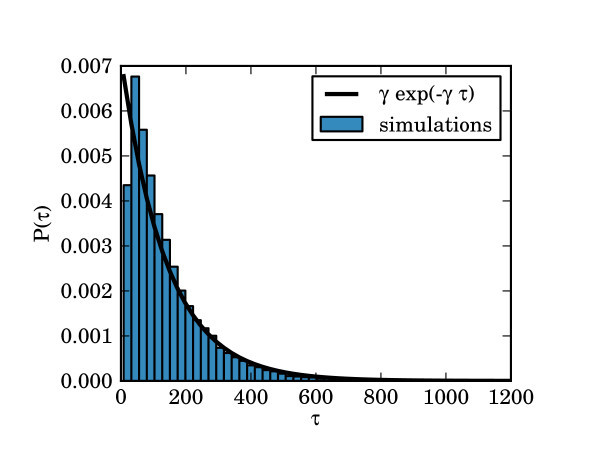
**Illustration of directed paths and the FTC**. The first transitive component is coloured red in **(b, c)**. Since the graph in **(a) **is strongly connected, its first transitive component is the whole graph. **(a) **Illustration of directed paths. The purple arrows form a directed path from the purple node to the green node. The green arrows form a directed path in the other direction. **(b) **If a directed path exists from a node *A *to a node *B*, then we say that *A *≪ *B*, which is a partial ordering among the nodes in a graph. In this graph, *A *≪ *B *is true for each pair of blue nodes, and also for each pair of red nodes. It is also true that *A_r _*≪ *B_b _*for each red node *A_r _*and each blue node *B_b_*. However, it is not true that *A_b _*≪ *B_r _*for any blue node *A_b _*and red node *B_r_*. The red nodes, thus, form a minimum for the partial ordering and are the FTC. **(c) **The first transitive component may also be disconnected. The definition of the FTC ensures that it is either strongly connected as in **(a, b) **or disconnected as is the case in **(c)**.

We have explored the relationship between noise, network structure and escape time in a phenomenological model of seizure initiation and have been able to explain the relationship between asymptotic escape times and the FTC of low-dimensional networks of 2, 3, or 4 nodes. We can summarise our main findings as follows. When coupling is weak (small *β*), all networks of a given size (including disconnected networks) have similar escape times. With intermediate coupling strengths, the number of connections in the network is a significant factor in determining escape times; networks with more connections have greater escape times. When coupling is strong, the escape time depends only on the *FTC *of the network. Figure 12 depicts the relationship between escape times, network size and topology in this strong coupling case. We found that the most significant property is the number of nodes *n *in the FTC. This means that, for a given network size *N*, strongly connected networks have greater escape times than weakly connected networks. Among networks whose FTCs are of the same size, *balanced *strongly connected networks have the greatest escape times. The escape times for these networks scale exponentially in *N*, the size of the network. The smallest escape times, for any given size of network, occur when the FTC consists of a single node. The escape time for these networks is constant in the network size *N*. All other networks come between these two extremes, which diverge as *N *increases. The particular value of the escape time for these networks appears well described by Equation 5.

Extending these findings to larger scale networks, inferred from EEG recordings, has enabled us to determine a statistically significant difference between escape times in networks associated with patients with IGE and those networks associated with controls. We interpret escape times as being inversely related to the frequency of occurrence of seizures. The result, then, is that we have found differences in 'brain connectivity parameters' in patients that are associated with a greater likelihood of having seizures in our simplistic model.

Our study raises a number of questions. First, why do we observe significant results in the high (beta and gamma, ≥ 15 Hz) frequency bands? It might be considered that since the dominant band in most epileptiform EEG is at a lower frequency than this, then we might expect to find significance in lower bands instead? However, the frequency of activity that underlies seizure initiation need not be in the same frequency band as the evolving seizure. The model used here assumes that white noise initiates seizures which then occur at approximately 3 Hz.

Second, our patient group is heterogenous, by which we mean they take different medications and experience different frequencies of seizures. A natural next step to extend our study would be to examine more homogenous groupings of patients, for example to examine the effect of successful versus unsuccessful treatment. A further extension would be to examine correlations between network structure and seizure frequency on a patient by patient basis.

Third, the normal group displays a non-zero seizure rate which might be considered a practical failing of the model. It is important to note that seizures can emerge in otherwise "normal" individuals in many situations where there is an acute disruption of normal brain function. For example in association with various drugs, alcohol or head trauma. Thus, an underlying predisposition to seizures may well be "normal" but is balanced by protective mechanisms (which we do not model within our phenomenological framework), which prevent seizures occurring normally. Mathematically, this is equivalent to the distance in phase space of the inter-ictal and ictal attractors being much greater in normal subjects, but both still exist (as suggested by Lopes da Silva et al. [15]). The conclusion of our present study is that the rate of seizure occurrence in our phenomenoloigcal model is much greater in patients than normals, in keeping with this. Similarly, many "'normal" people have a single seizure, but of those who have a single seizure, are neurologically normal and have apparently normal EEG, only 25% will have a second seizure (i.e., will be found to have epilepsy [21]). From either argument, it could therefore be postulated that seizure risk is indeed non-zero in "normal" subjects.

Finally our observation-that escape times are smaller in networks from the patient group for certain frequency bands-is suggestive that network structure may play an important role in determining seizure initiation and frequency. Any difference in network connectivity is likely to be associated with genetic factors, as is idiopathic generalised epilepsies themselves. Consequently, a natural extension of this research would be to apply this methodology to first-degree relatives of epilepsy patients.

## Appendix A (Calculation of escape time)

Recall that we identified *seizure frequency *with escape times of the model. Thus, in this appendix, we seek to write down an analytic expression for the escape time of our model (1).

## Method

The exit problem for an autonomous system can be stated as follows [22]. First, we must define an initial value problem, characterised by an Itô-style autonomous SDE,

(6) dx=a(x)dt+B(x)dw(t),

where **a**(**x**) and **B**(**x**) represent the drift and diffusion coefficient, respectively, d**w**(*t*) is a multidimensional Wiener process. The initial condition at time 0 is represented by **x**_0_. The exit problem concerns characterising the distribution of escape times, that is, the times taken to leave a chosen region of phase space. We can define the first escape time of a trajectory *τ***_x _**from a region **Ω **as

$$\tau_{x}=\text{inf}\left\{t\geq 0|x\left(t\right)\in \partial \Omega ,\;x\left(\text{0}\right)=x_{0}\right\},$$

where ∂**Ω **is the boundary of **Ω**. The subscript, **x**, indicates that the distribution of escape times depends on the choice of initial condition. To characterise the full distribution of escape times is difficult in general, but the *expectation *of the escape time as a function of initial condition can be calculated as:

$$E\left[\tau_{x}\right]=u\left(x\right),$$

where the function, *u*(**x**), is the solution to Dynkin's equation [23]:

(7) $$\begin{array}{llll} M_{\left[a,B\right]}u\left(x\right)\; & =\;-1,\;x\in \Omega \; & & \;\\ u\left(x\right)\; & =\;0,\;x\in \partial \Omega ,\; & & \;\\ & \; & \end{array}$$

where **Ω **is the region of phase space contained with the escape boundary ∂**Ω**. Here, the operator **M**_[**a**,**B**] _gives the infinitesimal generator for the system and incorporates the vector fields of the SDE,

(8) $$M_{\left[a,B\right]}u\left(x\right)=a\left(x\right)\cdot \nabla u\left(x\right)+\underset{i,j}{\sum }\sigma_{ij}\left(x\right)\frac{\partial^{2}u\left(x\right)}{\partial x_{i}\partial x_{j}},$$

where

$$\sigma \left(x\right)=\frac{1}{2}B\left(x\right)B{\left(x\right)}^{T}.$$

Unfortunately, Equation 7 can only be solved exactly in the case of trivial dynamical systems. However, Matkowsky and Schuss [24,25] provide results based on singular perturbation that remain asymptotically valid in the presence of small noise, for a restricted class of SDEs. Firstly, the function **a**(**x**)--representing the deterministic part of the dynamical system--must be expressible as the gradient of a scalar potential,

(9) a(x)=-∇ψ(x).

Secondly, the diffusion coefficient, **B**(**x**), must be constant and proportional to the identity matrix, *α***I**. This is equivalent to each equation in system (6) receives *additive *noise from its own *independent *Weiner process. Finally, the result is asymptotically valid only if the noise coefficient, *α*, is small. In general these are strong restrictions but in our case, the only relevant consideration is whether or not the noise is small enough.

We define *ψ*(**x**) as the potential function evaluated at point **x **in phase space and the system derivative is the (negative) gradient of this function. We assume that *ψ*(**x**) describes a system with a stable fixed point surrounded by a potential barrier and ask for the escape time over the barrier. $\hat{\psi }$ is the height of the potential barrier from the bottom of the well (at its lowest point around the boundary). **H**(**x**) is the Hessian matrix of second partial derivatives of *ψ *evaluated at the fixed point (assumed to be **x **= **0**). *c*(**x**) is half the absolute magnitude of the curvature of the potential function on the barrier in the direction normal to the barrier. Then Schuss's analytic result is that [25],

$$E\left[\tau \right]=\frac{\alpha^{n-1}\pi^{\frac{n+1}{2}}\text{exp}\left(2\frac{\hat{\psi }}{\alpha^{2}}\right)}{\text{det}|H{\left(0)\right|}^{\frac{1}{2}}\int_{U}\sqrt{c\left(x\right)}\text{d}S\left(x\right)},$$

where *n *is the dimensionality of the system and the integral is over *U *which is the subset of the points on the barrier at which *ψ *is equal to it's lowest value on the boundary (i.e., $\hat{\psi }$). Assuming that we can describe the system in terms of a suitable potential function, this equation allows us to immediately obtain, from the system definition, an approximate analytical expression for the escape time. The chief restriction on the validity of this approximation is the assumption that *α *is small.

Another approach to finding the escape times of dynamical systems under small noise is the Eyring-Kramer (EK) equation, which has been rigorously proved for multi-dimensional systems [26]. Like the Schuss result above, it can lead quickly to a formula for the escape time in terms of a potential function for the deterministic dynamics of a stochastic ordinary differential equation. However, although the formula exists and is proven for multidimensional systems, it requires that, in the singular limit, escapes take place almost surely at a finite number of discrete saddles. However, in the case considered above, escapes take place everywhere on a continuous arc of points even in the singular limit. To our knowledge, the EK formalism has not been used to develop an explicit formula in this case.

## Appendix B (Centre of mass dynamics)

Here, we consider the effect that linear, asymmetric, synchronising couplings has on centre of mass dynamics. In the absence of noise, the networked dynamical system presented above is of the form

$$\frac{\text{d}z_{i}}{\text{d}t}=f\left(z_{i}\right)+\beta \overset{N}{\underset{j=1}{\sum }}M_{ji}\left(z_{j}-z_{i}\right),$$

where **z***_i _*is the state of the *i*th node, *f *is the vector field for the isolated systems, *β *is the coupling strength and **M **is the normalised adjacency matrix describing the topology of the couplings between the nodes of the network. We define the centre of mass of this network as the mean state of the nodes,

$$\left\langle z_{i}\right\rangle =\frac{1}{N}\overset{N}{\underset{i=1}{\sum }}z_{i}$$

where *N *is the number of nodes in the network and 〈·〉 denotes the mean over all *i*. We have the equation of motion for the centre of mass of the network

$$\frac{\text{d}\left\langle z_{i}\right\rangle }{\text{d}t}=\left\langle f\left(z_{i}\right)\right\rangle +\frac{\beta }{N}\overset{N}{\underset{i=1}{\sum }}\overset{N}{\underset{j=1}{\sum }}M_{ji}\left(z_{j}-z_{i}\right).$$

The summation in the second term can be rewritten

$$\begin{array}{llll} \overset{N}{\underset{i=1}{\sum }}\overset{N}{\underset{j=1}{\sum }}M_{ji}\left(z_{j}-z_{i}\right) & =\overset{N}{\underset{i=1}{\sum }}\overset{N}{\underset{j=1}{\sum }}M_{ji}z_{j}-\overset{N}{\underset{i=1}{\sum }}\overset{N}{\underset{j=1}{\sum }}M_{ji}z_{i}\; & & \;\\ & =\overset{N}{\underset{i=1}{\sum }}\overset{N}{\underset{j=1}{\sum }}M_{ji}z_{j}-\overset{N}{\underset{i=1}{\sum }}\overset{N}{\underset{j=1}{\sum }}M_{ij}z_{j}\; & & \;\\ & \; & \end{array}$$

where we have exchanged indices in the second summation term. We can then recombine the two summation terms to find

$$\begin{array}{llll} \overset{N}{\underset{i=1}{\sum }}\overset{N}{\underset{j=1}{\sum }}M_{ji}\left(z_{j}-z_{i}\right) & =\overset{N}{\underset{i=1}{\sum }}\overset{N}{\underset{j=1}{\sum }}\left(M_{ji}z_{j}-M_{ij}z_{j}\right)\; & & \;\\ & =\overset{N}{\underset{j=1}{\sum }}\overset{N}{\underset{i=1}{\sum }}\left(M_{ji}-M_{ij}\right)z_{j}\; & & \;\\ & =\overset{N}{\underset{j=1}{\sum }}u_{j}z_{j}\; & & \;\\ & =u\cdot z,\; & & \;\\ & \; & \end{array}$$

where **u **is the vector such that

$$u_{j}=\overset{N}{\underset{i=1}{\sum }}\left(M_{ji}-M_{ij}\right)=\overset{N}{\underset{i=1}{\sum }}M_{ji}-\overset{N}{\underset{i=1}{\sum }}M_{ij}.$$

We can now rewrite the equation of motion for the centre of mass as

(10) $$\frac{\text{d}\left\langle z_{i}\right\rangle }{\text{d}t}=\left\langle f\left(z_{i}\right)\right\rangle +\frac{\beta }{N}u\cdot z.$$

For the system described above, *f *always points towards the fixed point, **z **= 0, within the escape boundary. The first term in Equation 10 represents the synchronised dynamics of the network and alway points towards 〈**z***_i_*〉 = 0. The second term, however, affects the dynamics of the network when the nodes are unsynchronised and can cause the centre of mass to move away from the fixed point provided **z **has a positive component in the direction of the vector **u**.

The *j*th element of the vector **u **is the difference between the *j*th row and column sums, respectively, of the network adjacency matrix. That is, **u***_j _*is the difference between the out-degree and the in-degree of the *j*th node in the network. Those nodes *j *for which **u***_j _*is positive are the nodes which are more able to draw the centre of mass of the network towards themselves, and therefore have a greater influence over the network as a whole than the other nodes. These nodes are those with more outgoing connections than incoming connections.

In the event that the adjacency matrix, **M**, is symmetric, the vector **u **will always be zero. This corresponds to the case in which all connections between pairs of nodes are bidirectional, or all those networks whose connectivity graph is equivalent to an undirected graph. However, this is not the only case in which the vector is zero; it will also be zero in the case of a network whose connectivity graph is a cycle graph or consists of a union of disjoint cycles. It is this analysis that lead to the concept of a *balanced graph*: a graph in which each node has the same out-degree as in-degree, and thus has **u **= 0.

## Appendix C (Definition of the FTC)

In the case of weakly connected graphs, we find that the escape behaviour of the network model considered above depends only on a particular subgraph that we call the *FTC*. In this appendix, we define what precisely is meant by the FTC. For any strongly connected graph, the FTC is the whole graph. Any weakly connected graph has a number of strongly connected components arranged in a hierarchy.

Intuitively, we can say that the FTC is the subgraph comprised by those strongly connected components at the top of the hierarchy. The concept of the FTC is illustrated in Figure 12, and also in Figure 7 which distinguishes the FTC of each weakly connected graph of three nodes.

We consider a *directed graph ***G **of *N *nodes (*A*, *B*,...). In a directed graph, an edge between two nodes, *A *and *B*, has a direction, either from *A *to *B *or from *B *to *A*. We denote an edge from a node *A *to a node *B *by *A *→ *B*. A *directed path*, in **G**, from a node *A *to a node *B *is a set of edges leading from *A *to *B*, possibly through other nodes as depicted in Figure 12a. For example, the edge set (*A *→ *B*, *B *→ *C*, . . . , *Y *→ *Z*), form a directed path from node *A *to node *Z*. We consider that there is always a trivial directed path from any node *A *to itself.

We can use the concept of a directed path to define a partial ordering relation between nodes within a directed graph. We say that *A *≪ *B *if there exists at least one directed path from *A *to *B*. There always exists a trivial directed path from any node to itself so that *A *≪ *A *is true for any node *A *in **G**.

The relation, *A *≪ *B*, defines a partial ordering of the nodes in a directed graph **G**. Consider the set **S **of all nodes that achieve the minimum for this ordering. The subgraph **G' **corresponding to the set **S **of minimal nodes in **G **is the *FTC *of the graph. For any strongly connected graph **G**, the FTC **G' **is equal to the whole graph **G**. For any weakly connected graph **G**, the FTC, **G'**, is a subgraph of **G**. The FTC must either be strongly connected or disconnected as in panels (b) and (c), respectively, of Figure 12 and *cannot *be only weakly connected.

## Appendix D (EEG collection and processing)

Thirty-five patients with *IGE *participated in the study, along with 40 healthy controls. Scalp electrodes were placed at locations FP1, FP2, F7, F8, F3, F4, FZ, T3, T4, C3, C4, CZ, T5, T6, P3, P4, PZ, O1, O2, A1, A2 in the modified Maudsley configuration [27], a variant of the standard 10-20 system in which the outer electrodes are positioned slightly lower, to improve coverage of deep temporal lobe structures in epilepsy patients. Data were recorded using a NicoletOne recording system (Viasys Healthcare, San Diego, California, USA), with open filters and a sampling rate of 256 Hz, referenced to an extra, midline electrode. Offline, channels A1 and A2 (the left and right earlobes) were excluded, and then the data were changed to an average reference montage. For analysis, a single 22nd data epoch during which subjects were sitting still with their eyes closed and which was uncontaminated with epileptiform or other artefacts such as movement or eye-blinks was extracted. This was bandpass filtered in the range [0.5, 70] Hz, and then notch filtered at 50 Hz. For the analysis, we consider here a single twenty-second epoch was extracted during which subjects were sitting still, eyes closed and the EEG was uncontaminated with SWD or other artefacts.

The resulting EEG timeseries were separated into five different frequency bands, delta (1-3 Hz), theta (4-8 Hz), alpha (9-14 Hz), beta (15-30 Hz), and gamma (31-70 Hz) bands. With 75 subjects and five frequency bands there were, in total 375 different timeseries. The Hilbert transform was then applied to the time series to generate instantaneous phase and amplitude estimates. A convenient measure of phase-locking can then be generated by estimating for each time point the phase difference between oscillations at a particular frequency recorded in two separate locations and calculating the absolute value of the mean of these phase differences considered as complex numbers with unit modulus. This is often referred to as the phase-locking factor (PLF) [28,29].

Precisely, we have two signals *X *and *Y *represented as digitally sampled signals with samples *x_i _*and *y_i _*for 1 ≤ *i *≤ *N*. We compute the discrete Hilbert transform of both signals giving the complex coefficients $x_{i}^{H}$and $y_{i}^{H}$ with 1 ≤ *i *≤ *N*. These coefficients are normalised to have unit modulus so that we have ${\hat{x}}_{i}^{H}=x_{i}^{H}/|x_{i}^{H}|$ and ${ŷ}_{i}^{H}=y_{i}^{H}/\left|y_{i}^{H}\right|$. The PLF between the two signals *X *and *Y *is then given by

(11) $$\text{PLF}\left\{X,Y\right\}\dot{=}\frac{1}{N}\overset{N}{\underset{i=1}{\sum }}\left|{\hat{x}}_{i}^{H}-{ŷ}_{i}^{H}\right|$$

The matrix **M **referred to above has entries **M***_jk_*, representing the PLF between the *j*th and *k*th EEG channels. Treating this matrix as a matrix Pearson correlation coefficients, we derive the matrix of *β*-weights from **R **= **M^-^**^1 ^by *β_jk _*= -**R***_jk_*/**R***_jj_*. The smallest entries in the matrix of *β*-weights were pruned until the non-zero entries of the matrix represented a graph with mean degree, *d*, where different values of *d *were used in different cases. This procedure results in an adjacency matrix of 0 and 1s representing an unweighted, directed, asymmetric graph.

## Competing interests

The authors declare that they have no competing interests.
